# Internship Experience: A Transition from Academic World to Health Care Workplace

**DOI:** 10.31729/jnma.7383

**Published:** 2022-03-31

**Authors:** Rupa Bhandari, Krity Basnet, Krishna Bhatta

**Affiliations:** 1 Kathmandu Medical College and Teaching Hospital, Sinamangal, Kathmandu, Nepal

**Keywords:** *internship*, *medical student*, *work*

## Abstract

Internship is the training period during MBBS in which medical students will be able to learn methods/modalities to administer actual practice of medical and health care based on the knowledge gainded in their medical school. The students will get short-term supervised professional learning experience in different departments of medical and surgical specialities and have opportunity to develop professional networks within their intended career field. Internship gets your foot in the door of the real-world health care practice environment from the theoretical learning. Gaining knowledge and skills by reflecting on mistakes in practical scenario will boosts confidence and refine the practice later on. An internship can be a powerful medium for personal and professional growth as it allows the sense of responsibility towards the patients, their family and the organisation. It is indeed a great opportunity to have a hands-on learning experience prior to graduation of medical school.

## INTRODUCTION

An internship during undergraduate study is a supervised training where a student is under supervision during the patient care instead of working alone as a independent doctor.^1^ The internship is a transition from the academic world to the working environment. Traditional education management has prepared students for functional specialities but had difficulty in developing student's practical skills, confidence, and insight into the real working environment. Social work education has recognized the importance of internship since 1893 and has been a mandatory part of education management.^2^ Internship experience gives exposure to practical skills, improves social relationships, motivates future learning and clearer view about the future, and enhances their social personality skills.^1,2^

## THE INTERNSHIP

The questions that comes into one's mind regarding internships are: why are internships important during medical school? why do students do internships? The answer to these question is that they are a short-term practical experience that provides opportunities to the students to enter real-world work field during and after their under-graduation course programs.^3^

Internship is a type of experiential learning that combines classroom knowledge and theory with real-world application and skill development in a professional setting. Internships are a way to complement your education and secure your future career. Internship provides a great insight into what it's like to work for a living, and gives an idea of how our health sector operates. The importance of internship experience comes from the skills you gain and the improvements you make to have a strong resume.^4^ Experiences in higher education is a crucial for personal growth, meeting new people and broadening your horizons, and experiencing life from a different viewpoint, but it is ultimately about preparing for a career path and your life beyond that.^4^

Being an undergraduate medical student in Medical College for 4.5 years, we started my internship in the same college after finishing final year. A medical intern is someone who has completed their medical school and practises medicine under supervision for a year as they don't have a full licence to practise independently. The internship has a certain amount of allocated credit hours.

Our internship period is of 365 days. Our internship period was divided into two majors: six months in surgery (Orthopaedics, Gynaecology and Obstetrics, Neurosurgery, Plastic Surgery, and Anaesthesia) and the remaining six months of medicine (Ophthalmology, Ear, Nose and Throat, Emergency Medicine, Internal Medicine).

The internship program began with an orientation class which covered aspects of medical ethics, techniques of sample collection for investigations, rational drug prescription, operation theatre protocol, medico-legal aspects, health research methodology, etc. A logbook was given to us and our progress is monitored through a logbook.^5^

## BEGINNING OF POSTINGS

Everything in an internship is new, the work, the people, and the culture. This is going to feel hard at first but when in doubt we can ask questions.^6^ At the start, we were posted in different departments, each department for one to two months where we were briefed and supervised by a mentor. During the shadowing period, we were briefed about the case, how to proceed the case and plan the treatment and outcomes. We got the opportunity to gain first-hand experiences in the day-to-day procedures. It was scary during the initial phases when you did not know where to put your hands and feet. However, as we dealt with the patients, we noticed that we became more relaxed and natural. Our apprehension of committing errors and embarrassment vanished after we acquired a new perspective from the experience.^7^

In this period, we learned how things proceed in a department. We had to be there at 8:30 AM every morning and ready to have positive interactions with patients. We obseve if there are any issues regarding the cases and go through the progress about patient's health, and report to the mentors during the ward round.

## INCORPORATION OF UNDERGRADUATE KNOWLEDGE AND SKILLS

During the internship, we were able to learn practical aspects of health care than what was tought through textbooks. The things which we learned during our academic period shave limited application to what we do in an internship. Another thing we learned during the internship is that we need to be well-versed on the most frequent ailments that affect our area. We are fascinated by unique cases and disorders during our undergraduate years, but later we realise it as a common ailment that we must concentrate on and conquer. Aside from theory, the hours that we invest in our clinical classes talking to the patient, taking their history, clinical examination, and looking at their charts and management also prepare us in some way for this transition into practical world.

## JOB RESPONSIBILITIES

In our medical school, we had to attend the outpatient department, make a community visit along with mentors. That program included health education sessions, health examination, screening for various diseases. During our internship, we did a few case reports, research articles. These all activities were closely supervised by faculty from each department.

The patient's progress and deterioration of health all depend on the team of health care providers of the hospital and we are also a part of that team. We learned to be vigilant and agile while dealing with patients. Sometimes, even the same case diagnosis needs to be dealt differently. An intern's job is not only to study but to prepare for their path. Studying along with work is a must during the internship.

## MAKING MOST OF IT

In experiential learning and internships, real learning comes after the work. A famous lesson from Confucius around 450 BC is, “tell me, and we will forget. Show me, and we may remember. Involve us, and we will understand".^8^ One learns to be independent and confident as the internship goes on.

We need to make the internship period worthwhile but to make the most of it, it is important to understand what aspects needs to really focused on. We need to have a clear understanding of what we want to learn as an intern where we want to go and achieve. Take ownership of duties, projects, showcase your abilities and contribution to the group. In the beginning, we weren't sure what we wanted to get out of this internship program or what we wanted to do afterward. However, it's quite acceptable to be clueless at first. We just simply enjoy the internship time and be attentive for the new learnings and experiences. Later on we explore ourselves, find our interests, strengths, weaknesses, not only in their knowledge or skills but also in behaviour and mindset. Setting continuous goals, and keeping track of progress and improvements is very important.^7^

Time management skills was learned during the internship with the addition of responsibilities. Writing articles, case reports, research papers, building a firm base for the future needs are advisable to start during internship periods. During the internship everyone is competitive and wanted to take ownership of duties, wants to have a first-hand procedure. There was “we”, we wanted to do it, everyone wanted to seek the opportunity and make the most of it. Having a competitive behaviour is important for improving yourself. We seek the opportunities, recognize it and improve ourself from the experiences. While being on internship, interns will also have the opportunity to discover their field interests and objectives under a professional mentor.^2^

## CHALLENGES OF INTERNSHIP

Internship at times was hard, stressful, and with a shortage of time. We made a lot of mistakes, blunders, repeated those mistakes but tried my best not to repeat the same. We worked in a different department every month and wasn't familiar with the working process. Every time we joined a new department we used to be nervous and quiet. We were afraid to ask questions because we thought that would make us seem dumb.^7^ We were given duties but was hard to complete duties on time. The trivial works that were given to us were not satisfying. At times, we don't feel the motivation, we thought we were not supposed to do that and they wouldn't get us anywhere.

There were times, when our work used to go unnoticed, in which we put our utmost effort. Sometimes we feel low and upset and didn't feel like doing things with the same efforts and enthusiasm. During internship, everyone wants to acquire a new skill, receive firsthand experience, and take ownership of tasks during an internship because this is the moment to show off your abilities and contribution to the group.

We often become quite nervous and afraid of doing something inappropriate in unfamiliar settings. University is a place to make mistakes. One should always ask questions no matter how stupid they might sound because it's always better than doing it in the wrong way and wasting everybody's time.^7^ Even simple procedures handling alone were difficult for the first time. One tends to get nervous, and being failed in the procedure during the learning period is always normal. It was okay not to able to perform 100 percentage. Procedures are not supposed to be known but are skills that need to be practised and get better with repetition.

Constructive criticism from mentors could help us grow and adapt to the work setting. Having an uncooperative mentor during an internship is always challenging. Knowing not only about the job (task significance, feedback) but also about the workplace (learning opportunities, supervisor support, and organisational support) and workpeople are an important key for making internships satisfying in any field.^9^

## PERKS OF INTERNSHIP

Another important part of the internship is to become financially independent and we expect that the organisation should pay us as regular employees. But interns and employees are different and administraters are not willing to pay as regular employees. Interns should be assigned duties that are appropriate for college students.^10^

Intellectual and emotional growth during internship are important for any aspects of life. To analyze information critically and think creatively are essential abilities. Communication skills, solving problems, working in a team will make comfortable both at home, work and community. It is an opportunity to become socialised into the norms and values of a profession. The internship is a summit of an academic experience in a highly structured and sequenced set of experiences.^11^ We developed friendships with our seniors, got feedback from our seniors and juniors, dealt with cooperative and non-cooperative patients, and treated some patients. The solidarity between us interns, and heading out together to socialise at the end of the exhausting day, made it all worthwhile.

## WAYS FORWARD

Practical skills and learning comes from the experiences. Our internship period in medical college allowed me to evolve and mature personally. It also helped me to gain skills in procedure/modalities and develop effective communication skills during patient care. We also gained a better understanding, and real-world experience and gained a few new references and a clearer view for the future. We could take the opportunity to do an internship for smooth transition from academic life to a real-life working environment even if it is not required in the field that you wish to work in. There is much to gain from it on both a professional and personal level. The completion of the internships program helps to make a clearer view of individual career, own efficiencies, weaknesses and strengthens. These Internship experience makes a college graduate more marketable as they usually require less training and can handle more responsibilities.
